# Current Landscape of Coccidioidomycosis

**DOI:** 10.3390/jof8040413

**Published:** 2022-04-17

**Authors:** Ryan Boro, Prema C. Iyer, Maciej A. Walczak

**Affiliations:** 1 Department of Pharmaceutical Sciences, University of Pittsburgh, Pittsburgh, PA 15213, USA; rrb51@pitt.edu; 2 Department of Chemistry, University of Colorado, Boulder, CO 80309, USA

**Keywords:** antifungal agents, coccidioidomycosis, *Coccidioides* spp., fungal co-infections

## Abstract

Coccidioidomycosis, also known as Valley fever, is an endemic fungal infection commonly found in the southwestern parts of the United States. However, the disease has seen an increase in both in its area of residency and its prevalence. This review compiles some of the latest information on the epidemiology, current and in-development pharmaceutical approaches to treat the disease, trends and projections, diagnostic concerns, and the overlapping dynamics of coccidioidomycosis and COVID-19, including in special populations. This review provides an overview of the current diagnostic and therapeutic strategies and identifies areas of future development.

## 1. Introduction

**Figure 1:**
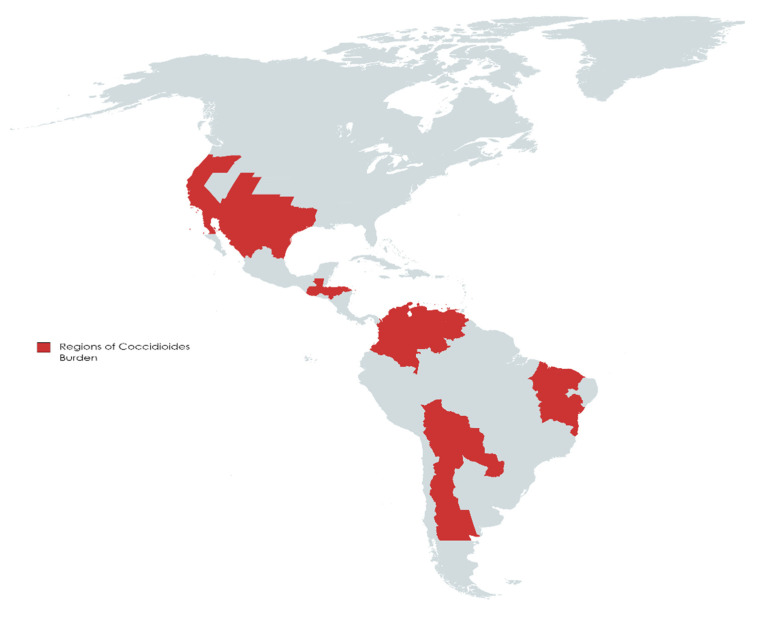
Regions of *Coccidioides* burden. Regions in North America have a longer history of surveillance than Central and South America. It is suggested due to the discrepancy that the impact of CM may be underestimated in Central and South America [5,6,7]. Figure created with MapChart.net.

Coccidioidomycosis (CM), also known as San Joaquin Valley fever or Valley fever, is a fungal disease endemic in certain parts of the Unites States, predominantly in California and Arizona [1,2]. Valley fever is caused by *Coccidioides*, a dimorphic fungus, first described in 1892 [3]. Since 2002, it has been recognized as two separate species, including the previously categorized *C. immitis*, and the newer designated *C. posadasii* [4]. *C. immitis* mainly resides in California, Washington State, Arizona, and Utah, while *C. posadasii* is mostly found in Arizona, New Mexico, Texas, Northern Mexico (Baja California, Chihuahua, Nuevo León, Sinaloa, Sonora, and Tamaulipas), and parts of Central (Guatemala and Honduras) and South America (Northern and Central Argentina, Bolivia, Colombia, Northeastern Brazil, Paraguay, and Venezuela) [5,6,7,8] (Figure 1).

**Figure 2:**
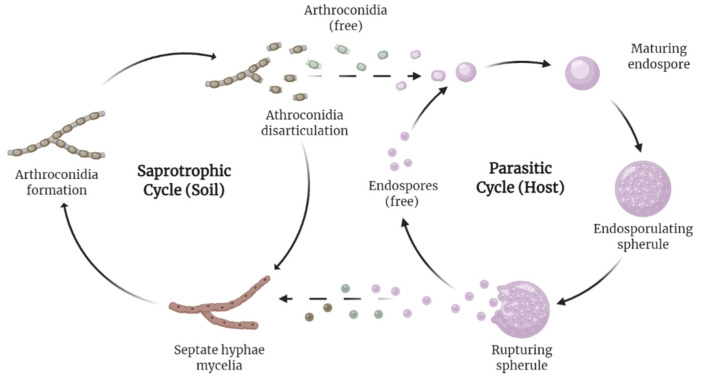
Life cycle of *Coccidioides*. The fungus possesses a dimorphic life cycle, living saprotrophically in soil and parasitically in its host at different intervals of the cycle. Recently, an alternative hypothesis formed, suggesting the species may be endozoan.

The *Coccidioides* spp. is a dimorphic fungus that grows as a mold in the environment at temperatures below 37 °C, and as a spherule in the host (Figure 2) [9]. *Coccidioides* exists as a saprotroph in the soil, feeding off decayed organic matter such as hyphae, and forms asexual spores known as arthroconidia. Soil disturbance distributes the arthroconidia into the air, and once inhaled by a living host, it converts to a parasitic state, ciphering nourishment from the host and forming endospores. The endospores eventually transform into spherules containing their own endospores, and, once ruptured, spread their contents to restart the parasitic cycle in the host, and possibly re-enter the soil in the environment [10]. More recent findings, including genomic analysis [11], support an alternative hypothesis, where the species acts as an endozoan living in mammalian hosts without causing detectable disease. Then, once the host dies, the organism establishes itself in the environment, utilizing its prior host’s dead biological matter to flourish [12]. Some mammals, such as dogs [13], appear to be reservoirs of the disease [14], but no evidence exists for zoonotic transmission [1].

## 2. Burden and Projections

Trends and projections reveal a situation where CM appears to be an increasing concern. Case occurrence increased immensely, moving from an age-adjusted incidence of 5.3 cases in endemic areas in 1998 to 42.6 per 100,000 in 2011 [15]. Cases may be underreported, with only 22 U.S. states having the disease reportable. It is suggested the true numbers of symptomatic cases are 6 to 14 times greater than what is reported to public health authorities [16]. While, as stated previously, the disease resides mostly in CA and AZ, more recent evidence of local infection in Washington [17] and environmental demonstration in Utah appeared [18]. The first documented case of CM in Africa occurred in a patient without a prior history of traveling outside of Uganda [19]. Latin America poses an emerging phenomenon for CM as the area historically contains an underserved population, surveillance of the disease is poor, and the numbers are observed less than they are officially recorded [20]. Much of the research done geographically exists in the areas of Mexico bordering the United States [20]. In Central America, the arid and semiarid countries of Guatemala and Honduras possess the largest presence of CM relative to the rest of the area [20]. Within South America, Brazil, with its established resources geared towards mycology research, contains some of the highest reported CM data relative to its neighboring nations [20]. The endemic areas observed in Argentina, while large in size, contained a small number of cases before the year 2000, with 63 of the 128 documented cases of CM occurring after 2000 [6].

Coccidioidomycosis manifests itself in the human population across the spectrum of clinical severity. Patients may present as asymptomatic in its mildest form, trending worse in presentation as pneumonic, pulmonary, fibro-cavitary, and disseminated [15]. An estimated 60% of all CM cases are asymptomatic [15], and the number of infections per year has risen to approximately 150,000 (one-half to two-thirds being subclinical) [1]. Most patients are protected from secondary infections [1]. Out of these estimated cases, 50,000 likely produced an illness warranting medical attention, 10,000–20,000 are diagnosed and reported, 2000–3000 produced pulmonary sequelae, 600–1000 moved to disseminated infection (spreading beyond pulmonary), and 160 resulted in death [21]. Because the most common clinical syndrome resulting from infection is community-acquired pneumonia (CAP), proper early diagnosis proves difficult due to many possible causes of CAP [22]. Disseminated disease can occur in virtually any site of infection, but most commonly seen as osteomyelitis, synovitis, lymphadenitis, soft tissue infections, cutaneous disease, peritonitis, and meningitis [23]. Disseminated disease results in the most serious cases, and while the overall occurrence of disseminated cases appears <1% [24], the high-risk population could be as high as 15% [25]. High-risk population includes exogenous immunosuppression (i.e., steroids and biologics), pregnancy, certain racial/ethical groups, and specific genetic defects within the IL-12/IFN-γ axis and STAT3-mediated pathway (the last appears to be essential in the immune response against CM) [26]. Meningitis-associated disease includes some of the worst clinical scenarios, occurring in nearly one-half of cases of disseminated disease, and may appear rarely years after primary infection [27].

Even with the high occurrence of manageable disease, CM cost remains substantial. Almost 75% of patients miss work or school due to infection and 40% require hospitalization [16]. In California, the estimated total costs consist of $429 million in direct and $271 million in indirect cost for a lifetime of cases reported each year [28]. A projected financial burden tied to climate projections illustrated the potential financial impact of the disease [29]. The estimated current annual medical costs, lost income, and economic welfare losses in the United States are as high as $400,000 per case, and the annual average total cost is $3.9 billion per year. In addition, the total annual burden can increase up to 164% by year 2050 and up to 380% by 2090 when higher greenhouse gas predictions and population growth estimates are included.

It has been observed that changing climate can affect the ability of fungi to cause harm, growth in the areas of concern, and prevalence of the afflicting fungi [30]. A specific example is *Candida auris*, first identified as a drug-resistant fungus in 2009 [31]. The researchers proposed that, among other factors driving its ability to thrive, the increasing temperature associated with climate change select for fungi which are more tolerant of higher temperatures. Therefore, the fungi become better suited for the body of a human host. Climate concerns appear to be important for CM as well. Recent analysis suggested remapping the area stricken with endemic fungal infections associated with CM in addition to histoplasmosis, blastomycosis, paracoccidioidomycosis, and talaromycosis [32]. The authors ascribe the change to climate change, in addition to other hypothesized global factors of agricultural techniques, occupational hazard, forest erosion, human migrating patterns, and soil dispersion, medical aspects of immune suppressants, higher disease recognition, and better diagnostic tests. Another study suggested an increasing impact of climate change in CM infections in California [33]. To compound the problem, research suggested communicating climate and CM dynamics can be difficult in the state, partly due to political views in areas heavily impacted by CM correlating with climate change denialism [34]. In a series of primary cutaneous CM cases, the authors linked their occurrences in Southern California to climate change [35]. The cases of note happened after an extended drought period followed by heavy rainfall in areas of Orange County in 2016 and early 2017, a place not associated with the endemic disease. The authors concluded that the unusual weather pattern was the culprit for the infections, as it has been demonstrated previously how robust *C. immitis* can be in drought epochs in comparison to other fungi [36]. Doctors at the University of California San Francisco reported a similar circumstance of increased CM diagnoses around the same time, mentioning the largest amount of CM diagnoses in the state since 1995 per the CA Department of Health [37]. Combined climate and mammalian reservoir modelling, specifically rodents, concluded a future increase in habitat suitability for the *Coccidioides* spp. [36]. A recent climate projection analysis by Gorris et al. [38] painted a significant increase in the temperature of a suitable climate for *Coccidioides* residency in the United States in addition to increased case occurrence. The study found that climate patterns would increase the regions affected by CM northward by 2100 into the states of North Dakota, South Dakota, Idaho, Wyoming, Montana, and Nebraska, in addition to yearly CM case counts by 50%. Projections for CM would not be complete without mentioning recent statistical reports published to predict future caseloads [39].

With the uptrend in cannabis legalization in the United States over the past decades [40], it is important to assess its impact on the incidence of fungal infections, including CM. Many who use marijuana for medicinal purposes do so under a potentially immunocompromised situation, such as for pain, nausea relief, and/or appetite stimulation due to cancer, transplant, or HIV [41]. These circumstances leave the patient susceptible to many types of infections, including those linked to fungal contamination in cannabis [42]. In a study by Benedict et al. [43], the authors utilized the 2016 IBM MarketScan Research Databases to include claims of over 27 million commercially insured employees, dependents, and retirees throughout the United States to assess cannabis use and its effect on fungal infections, including incidence of CM. The study concluded that those who used cannabis were 3.5 times more likely to have a fungal infection than a control group who did not use cannabis. A limitation of the study includes the ability to source where the infection arose. However, it is important to continue vigilance and possibly monitor the trends of the potential cannabis and fungal infection link, particularly where less stringent cannabis regulations exist in the traditionally CM-burdened southwestern United States [40].

## 3. Diagnosis

Initial diagnosis of CM based on the signs and symptoms proves to be an issue because of its similarity to other lung diseases. Many people infected with CM have no or minimal symptoms, including mild to severe respiratory symptoms, joint pain, malaise, fatigue, and fever. If symptoms occur, they usually can be observed one to three weeks after exposure and can last from a few weeks to a few months. Patients infected with CM have a mild respiratory illness with infiltrates or can have pulmonary disease presenting as nodules, cavities, or fibrocaviatary disease. In immunocompromised or otherwise healthy but genetically predisposed patients, CM can present as disseminated disease [26,44]. Recently published cases with atypical presentations provided evidence of further diagnostic complications, and this included initial mimicry for testicular cancer [45], rare aortic infection [46], polyarticular septic arthritis [47], CM of the vocal cords [48], and septic shock with multiorgan failure [49]. A study by Pu et al. [50] assessed delayed CM diagnosis and utilized CM cases over three years within a large Arizona health care system. The group implemented a diagnostic manual to overtly assist primary, urgent, and emergency healthcare providers in detecting CM, employing data from two years prior and one year after the implementation. Overall, 72.9% of diagnoses were made during hospital admission, 21.7% in ambulatory clinics, 3.2% in emergency units, and 0.5% in urgent care units. The study concluded that a large number of hospital admissions, attendant costs, and unneeded antibacterial drugs would have been avoided if improvements could be made to initially diagnose the disease. The implemented program resulted in increase of positive tests, but the modest outcomes suggest that an alternative method may be better for improving the timing of initial diagnosis.

Another aspect of diagnosis centers upon the correct tools to ensure accurate CM identification. Historically, antibody testing remains the mainstay of CM diagnosis, with research aimed towards perfecting the test in addition to other tools such as PCR and antigen testing [51,52]. A report by Kassis et al. [53] is a retroactive study that was performed to evaluate a combined antigen and antibody testing for progressive pulmonary and disseminated CM. Testing for antigens, a method first introduced in 2007, may provide better sensitivity in situations where antibody testing fails [52]. The study assessed the antibody, serum antigen, and urine antigen testing and concluded the most accurate diagnosis consisted of a combination of all the above rather than each alone or other combinations of assessment. The paper ultimately highlights the need for further research for better protocols in diagnostic accuracy.

## 4. Currently Approved Drugs

**Figure 3:**
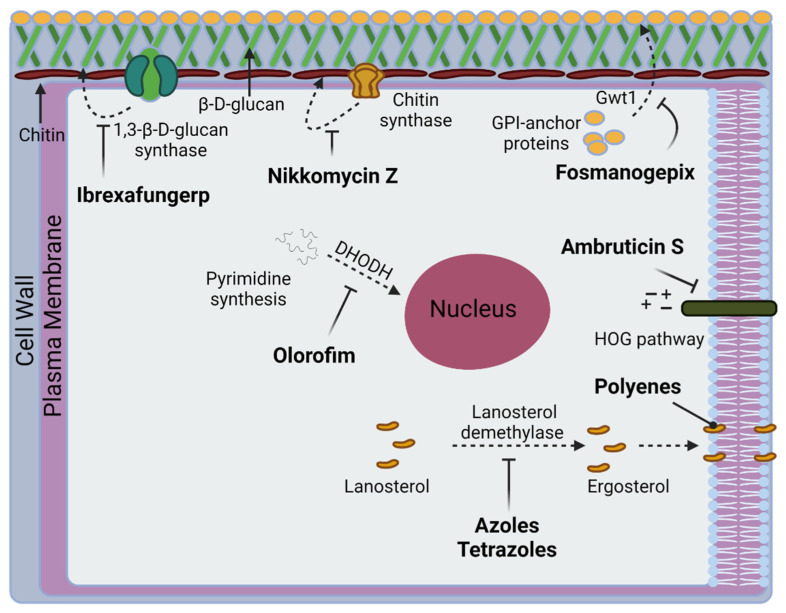
Fungal targets relevant to CM in current treatment, clinical trials, or preclinical development.

**Figure 4:**
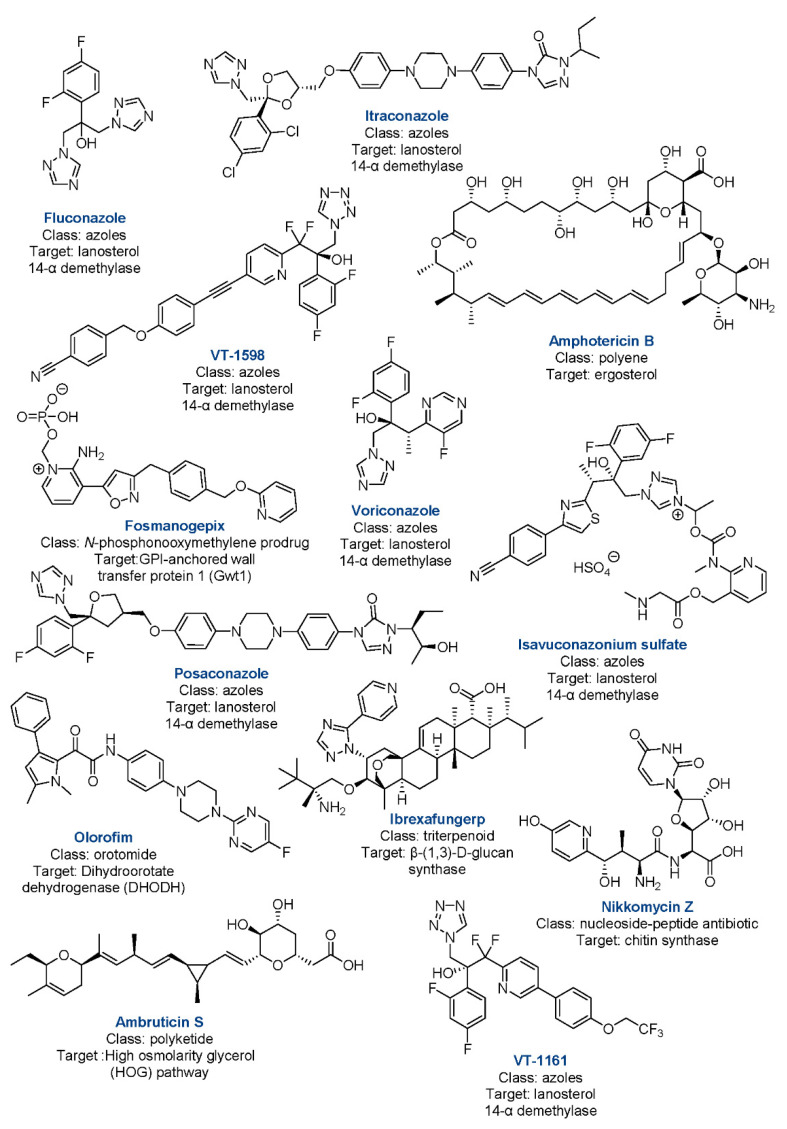
Drugs relevant to CM in current treatment, clinical trials, or preclinical development with class and targets.

A variety of compounds relevant in CM therapy at various levels of pharmaceutical development and targeting various pathways are known (Figure 3 and Figure 4). Current recommendations by the Infectious Diseases Society of America (IDSA) for antifungal treatment of CM consist of fluconazole or itraconazole as initial therapy and amphotericin B (AmB) for disease of a worse prognosis [54]. The evidence-based treatment guidelines for CM from the IDSA consist of a lower-quality variety when assessed on the Grading of Recommendations, Assessment, Development, and Evaluations (GRADE) scale [55]. Present FDA labeling reflects the statement, with the FDA citing only preclinical evidence for AmB usage in CM, while not mentioning the disease with fluconazole and itraconazole [56,57,58].

A. Azoles. The azole drugs act by inhibiting 14-α-demethylation of the CYP51 enzyme (present in humans and in the fungi), foiling the conversion of lanosterol to ergosterol [59]. Many adverse effects of azoles can be contributed to cross-inhibition of human enzymes such as CYP3A4, 2C9, and 2C19—all of which extensively metabolize other medications [59]. Furthermore, teratogenicity of this class of drugs can be contributed to CYP51 inhibition, leading to the recommendation of avoiding treatments in the first trimester of pregnancy [60]. Typically, first-line therapy consists of oral daily doses for patients with normal renal function [54]. Therapy may become prolonged or lifelong depending on the patient’s disease burden, clinical response, and host immune factors, with xerosis, alopecia, and fatigue being the most common adverse reactions [61]. Daily doses of itraconazole as the initial therapy are also recommended. However, sufficient absorption and increased chance of additional drug–drug interactions compared to fluconazole may be of concern [61].

Questions with respect to how best utilize additional azoles remain minimally answered, particularly in the face of evidence suggesting resistance to the first-line fluconazole [62]. Traditionally reserved for salvage therapy, voriconazole and posaconazole possess some positive evidence in the disease; however, no randomized controlled clinical trials addressing CM exist [59]. Both compounds have their limitations, with voriconazole demonstrating drug–drug interactions, bioavailability issues, and long term toxicity concerns, whereas posaconazole possesses poor CNS penetration and no proven clinical advantage compared to the less costly fluconazole and itraconazole [59]. More recent attempts were made to better understand the potential role of isavuconazonium sulfate (isavusulf) against CM. Isavusulf, a prodrug formulation of isavuconazole, is a second-generation triazole with activity against a broad spectrum of clinically important fungi [63]. Isavuconazole demonstrated potent in vitro activity against clinical isolates of *Coccidioides* [64]. Furthermore, a previous series of nine patients demonstrated improvement utilizing the drug as salvage therapy in refractory disseminated CM [65]. In a study by Kovanda et al., the researchers highlighted the lack of experimental modeling in isavuconazole in addition to recommended antifungals for CM [66]. This work attempted to address the problem by creating survival and pharmacodynamic-pharmacokinetic (PD-PK) murine models utilizing isavulsulf, fluconazole, and no treatment groups. The study demonstrated that isavulsulf and fluconazole caused a significant reduction in fungal burden in mice compared to the no treatment control in a dose-dependent manner. They also demonstrated that increasing exposure to the drugs resulted in decrease in fungal burden over time in a PK-PD model. In the end, the study provides much needed data in a preclinical CM experimental model that can guide future developments.

CM resulting in meningitis has some of the worst overall outcomes in the disease [27]. In addition, little data exists in the literature characterizing isavuconazole’s ability to concentrate in the cerebrospinal fluid (CSF). To characterize the concentration of isavuconazole in CSF, Davis et al. reported a study of treatment of refractory coccidioidal meningitis with concomitant cerebrospinal fluid and plasma therapeutic drug monitoring in a small case series of patients [67]. The study illustrated isavuconazole detectable in lumbar but not ventricular CSF. The authors concluded that a higher than standard isavuconazole doses might be required for better CSF penetration. This, in conjunction with meticulous management of intracranial pressure, might be required for adequate treatment of coccidioidal meningitis.

B. Polyenes. Considered as a member of the polyene macrolide class, amphotericin B (AmB) binds to ergosterol, a component of fungal cell membranes, and causes leakage of essential ions for the cell’s operation (such as Na^+^) leading to eventual cell death [68]. Although the compound has been in use since 1957 [69,70], it is characterized by noticeable toxicity, and manifests acute infusion-related reactions and dose-related nephrotoxicity [71]. AmB should only be reserved for the most serious cases, including failure of azole therapy and disseminated disease [54]. Liposomal formulations of AmB are effective in severe CM cases, but are also better-tolerated [72].

## 5. New Drugs in the Pipeline

Several promising compounds exist in the clinical trial pipelines addressing CM. VT-1598, a member of the tetrazole family, is currently in Phase 1 clinical trials for the treatment of CM (ClinicalTrials.gov Identifier: NCT04208321) [73]. The compound’s mode of action is similar to the other members in the azole family that achieve inhibition of 14-α-demethylase, however it is selective for fungal CYP51 enzymes over human ones [73]. Olorofim, the first member of the orotomide class, is making its way to clinical approval [74,75]. The compound acts as a reversible inhibitor of the enzyme dihyroorotate dehydrogenase (DHODH), an oxidoreductase that catalyzes de novo synthesis of pyrimidine [74,75]. First displaying evidence in murine experiment models for central nervous system CM infection, a Phase 2b clinical trial (ClinicalTrials.gov Identifier F901318) assessing the drug against resistant invasive fungal infections recently gained breakthrough status by the FDA in October of 2020 for “treatment of Central Nervous System (CNS) coccidioidomycosis refractory or otherwise unable to be treated with standard of care therapy” [76]. Ibrexafungerp, a first-in-class triterpenoid antifungal, acts as a noncompetitive inhibitor of the β-(1,3)-D-glucan synthase enzyme, resulting in the stoppage of synthesis of the essential fungal cell wall component β-(1,3)-D-glucan [77]. A Phase 3 trial assessing its use in fungal diseases that are refractory to or intolerant of standard antifungal treatment (ClinicalTrials.gov Identifier NCT03059992) expanded its investigated conditions to include CM. Another first-in-class molecule, fosmanogepix, currently is in Phase 2 trials with CM as a tested condition (ClinicalTrials.gov Identifier NCT04240886) [78]. The drug inhibits the Gwt1 enzyme which catalyzes inositol acylation—this is a preliminary step in the GPI-anchor biosynthesis [78]. Nikkomycin Z became of interest over the past decade in regard to a potential CM treatment [79]. The molecule inhibits chitin synthase, a foundational block in the cell walls of fungi [79]. Nikkomycin Z successfully completed a Phase 1 clinical trial [80] and was recently placed into murine modelling for preparation of as Phase 2 trials [81]. However, further clinical trials termed due to recruitment issues and lack of funding (ClinicalTrials.gov Identifier: NCT00614666). As of the time of this publication, no other current clinical trials exist for new entities treating CM. Of recent note, murine modelling for a sustained release formulation demonstrated efficacy against CM, negating one of the drawbacks of potential utilization in its short half-life [82]. Ambruticin S, a compound first discovered in the 1970s, proved promising in activity against *C. immitis* [83,84]. Its antifungal activity is attributed to the effect on osmoregulation via the high-osmolarity glycerol (HOG) signaling pathway [85,86,87,88,89]. Murine modelling of analogs of the compound verified in vivo activity against CM. However, neither this compound nor any of its derivatives have progressed into human trials. VT-1161 is another tetrazole that has demonstrated experimental efficacy in CM [90], including demonstrated efficacy in dogs [91]. While completed or ongoing clinical trials exist observing the conditions of recurrent vulvovaginal candidiasis, onychomycosis, candidiasis, and tinea pedis, none assessing use in CM presently exist.

To summarize this section, we note that while small-molecule treatment options may be sparse, the pipeline illustrate a promise of new tools to fight CM, available in the near future. Furthermore, some underexplored compounds may be a place for further development and can provide an opportunity for innovation. Orphan drug status candidates prove to be hard to fully develop, with CM not being one of the exceptions [92]. However, opportunities still exist for the scientific community to expound and optimize.

## 6. Immunological Therapies

Immunomodulating therapies against CM continue to be an underdeveloped area. Tsai et al. described a pediatric case of disseminated CM which resolved after interferon-γ and dupilumab therapy [93]. A treatment regimen of fluconazole and liposomal amphotericin B, surgical debridement, high-dose liposomal amphotericin B, posaconazole, and even as far as the antidepressant sertraline (with its evidence of in vitro activity against *C. immitis* [94]) failed in one reported case. Interferon-γ receptors, STAT1, STAT3, and interleukin-12 dynamics, all which contribute to susceptibility to disseminated CM, were tested, and impaired interleukin-12 activity became the deficiency of interest. An interferon-γ therapy demonstrated positive evidence in previous cases of disseminated CM [95,96] and aided in correcting the interleukin-12 deficiency. However, while the clinical progression slowed, disease resolution did not occur. This case demonstrates possible immunomodulating therapies to further investigate in use of CM and particularly disseminated disease. Furthermore, De la Hoz et al. [97] documented the first case of persistent CM resolved with voriconazole and interferon-gamma (INF-γ) adjuvant therapy. The first documented case of disseminated CM in a 16-year-old patient with chronic immunologic therapy for juvenile idiopathic arthritis (JIA) was also reported [98].

## 7. Vaccines

A vaccine against coccidioidomycosis is highly desirable because second infections are rare, thereby suggesting initial infections and possibly vaccinations confer life-long immunity [99]. Previous research projected that a CM vaccine would save 1.9 quality-adjusted life days and $62,000 per quality-adjusted life year, 11 fewer deaths, and $3 million annually [100]. An effort to create a formalin-killed spherule vaccine took place already in the 1980s; however, it did not provide a statistically significant response in clinical trials [101]. Research eventually turned towards exploring specific peptide vehicles [101]. In 2006, peptide vaccines utilizing antigens Pep1, Amn1, and Plb demonstrated evidence of inducting sufficient immune response in mice models [102,103]. These three antigens were then placed in an epitope base vaccine which has positive results in mice [104]. An additional vaccine vector displaying promise in murine models involved a recombinant chimeric polypeptide vaccine [105]. Recently, Powell et al. demonstrated the immunological value of a potential CM vaccine [106]. Employing knockout mice with various mutations hindering their susceptibility to disseminated CMs, such as STAT3 and IFNγ, the researchers tested a previously vetted vaccine vector, the avirulent strain of *C. posadasii* Δcps1 [107]. The group then challenged the immunodeficient mice in addition to a control with pathogenic *C. posadasii*. The vaccination mitigated the obstruction of most of the mutations explored. A canine-modelled vaccine candidate recently displayed promise, providing another avenue of potential work [108]. Research also focused on dendritic cell-based vaccines over the past few decades [101]. The research used the Ag2/PRA antigen found within the cell wall of *Coccidioides* [101]. Mice immunized with Ag2/PRA cDNA transfected into JAWS II dendritic cells were challenged with *C. posadasii*, and the vaccine increased positive outcomes versus the controls [101]. An intranasal version also met vetted criteria in murine modelling for cellular and humoral responses [101]. Detailing each attempt is outside the scope of this review; however, the formalin spherule vaccine stands as the only attempt to reach clinical development as of this writing [109].

## 8. CM and COVID-19

A review of systemic fungal mycoses in the era of the COVID-19 pandemic point to the challenge of COVID-19 (SARS-CoV-2) being seen as the exclusive reason for the patient’s condition, thereby removing suggestion of fungal infection causing the scenario [110]. Another way to refer to the situation would be the “anchoring bias,” defined as relying too heavily on a specific piece of information in order to make a decision, thereby creating situations of delay in treatment for the correct diagnosis experienced [111]. In general, it has been reported that COVID-19 did not impact testing frequencies for CM, histoplasmosis, blastomycosis, and cryptococcosis respiratory infections among a group of 174 infectious disease specialists; however, this observation should be taken with caution due to significant evidence of the diseases being historically underdiagnosed [112]. Furthermore, a lack of bronchoscopies and necropsies, occurring due to aerosolization risk of COVID-19, may be to blame for lack of fungal infection cases being reported [113]. 

A recent work highlighted the overlapping dynamics of COVID-19 and CM co-infection [114]. The study systematically examined the risk for co-infections among construction and agricultural workers, incarcerated persons, Black and Latino populations, and persons living in high-dust areas. Common risk factors for co-infection are age, diabetes, immunosuppression, racial or ethnic minority status, and smoking. Due to similarities in the symptoms between the two diseases, the COVID-19 pandemic might exacerbate delays in coccidioidomycosis diagnosis, potentially interfering with prompt administration of antifungal therapies. In chronic CM patients, increased susceptibility to COVID-19 is expected in people with compromised respiratory function. Also, reactivation of CM may occur with the increased immunological burden of COVID-19. Recent publications address specific instances of CM and COVID-19 overlap [115,116,117,118,119,120,121,122]. Two cases highlight dexamethasone usage [115,116,123] and poorer outcomes in CM prognosis, with one being fatal [115]. Previous research also suggests that dexamethasone without concurrent antifungal therapy may severely affect one’s fight against the disease [124]. The occurrence should suggest caution in intertwining drug therapies of both COVID-19 and CM. Immunosuppression has been tied to opportunistic infections in patients [125], and therapies becoming applicable in COVID-19 treatment, such as systemic corticosteroids, inflammatory cytokine antagonists, and Janus kinase inhibitors (JAKis) may lead to a rise in the opportunistic infections, including CM [125]. Systemic corticosteroids, established as a staple of COVID-19 therapy and confirmed by a recent meta-analysis, were connected to a lesser 28-day all-cause mortality in critically ill COVID-19 patients [126]. Potential overlaps in CM and COVID-19 therapy should be of concern. The JAKi tofacitinib used in COVID-19 therapy previously demonstrated marked increases in plasma levels when co-administered with fluconazole in half-life and AUC level as consequence of fluconazole’s inhibition of CYP3A4 [127]. COVID-19 guidelines suggest to adjust itraconazole therapy if co-administered with ritonavir-boosted nirmatrelvir [123].

In summary, similar presentations of both diseases create further difficulties in proper diagnosis of CM. The current clinical practice needs to account for a possible delayed diagnosis of CM and untimely antifungal treatments, particularly among clinicians in endemic areas of CM. Also, drug–drug interactions must be accounted for if COVID-19 and CM therapies are both conducted simultaneously.

## 9. Special Populations

Due to the relatively small size of overall population impacted by CM, discussions regarding specific clinical demographics remain sparse. Saling et al. attempted to address the intersection of CM and allogeneic bone marrow transplant (allo-HCT) recipients [128]. The study aggregated 21 cases, 2 presented by the authors and 19 from literature review, pertaining to the situation. The researchers concluded that the incidence of active CM in the population seemed low and can be attributed to regular use of antifungals as prophylaxis and post-transplant treatment. The same study emphasizes a detailed travel history and proper serological testing for *Coccidioides* before transplant and suggests antifungal prophylaxis of 100 days when applicable. Also, considerations of extending post-transplant antifungal treatment beyond the typical six months may be relevant if primary CM infection occurs. The study concluded that further study of various components of the full allo-HCT process and factors increase CM susceptibility, including engraftment phase, conditioning regimen, and prolonged neutropenia are needed. Lastly, it was suggested that a more data-driven inquiry into optimal duration of prophylaxis and treatment could improve therapeutic outcomes.

Vaugh et al. investigated another small but important population: neonatal coccidioidomycosis [129]. The study focused on three cases from a tertiary care children’s hospital in an endemic area, then performed a review from the past seven decades for additional information. The authors also found nine further cases relevant to this study in the literature. In many of the cases, symptoms presented within the first 1 to 4 weeks of life. However, diagnosis did not occur until months later; this is a concern, due to many cases displaying eventually extrapulmonary symptoms. Some patients initially tested negative for the disease but later converted to positive. Another concern is that antibodies may transfer from the mother at a point in time. However, additional clinical observation should further confirm the disease with positive serologic tests. One area of discussion that pertains is how the infant ultimately acquires the disease. The authors argue that it is unlikely the disease may transfer from mother to child via the placenta due to endospore size, but also acknowledge past research proposing the mother’s genital tract as a vector for transmission (specifically through aspiration of infected decidua during delivery). The study accentuated a rare circumstance where proper CM testing early leads to a decrease of disseminated disease. This work and a study by Naeem et al. [130] demonstrate the importance of proper diagnosis in endemic areas of CM, particularly with demographics as fragile as infants and pregnant women.

## 10. Conclusions

Our understanding of CM diagnosis and management continues to be a work in progress, and much of what has been implemented clinically is derived from historic precedence and observational studies. As the disease landscape continues to evolve due to climate change, the idea of “traditionally endemic” may need revision as well. Furthermore, the therapeutic tools supported by the evidence to fight the disease are limited, but new candidates that can add to our pharmaceutical arsenal against CM are on the horizon. These new candidates may open an opportunity for research to identify new targets and design novel drug entities, in addition to reinvestigate some of the compounds that demonstrated promise at the preclinical stage. Furthermore, the overlap between CM and COVID-19 must be acknowledged due to similar presentations, and proper diagnosis and management must be emphasized to avoid delayed CM diagnoses.

## Data Availability

Not applicable.
